# Torsion and clinical features in patients with acquired fourth cranial nerve palsy

**DOI:** 10.1038/s41598-024-58046-2

**Published:** 2024-03-27

**Authors:** Heeyoung Choi, Sang Yoon Kim, Su-Jin Kim, Jaehwan Choi, Seungahn Yang, Kwang Eon Han, Ji-Eun Lee

**Affiliations:** 1 Department of Ophthalmology, School of Medicine, Pusan National University, Biomedical Research Institute, Pusan National University Hospital, Busan, South Korea; 2 Department of Ophthalmology, Pusan National University Yangsan Hospital, Pusan National University School of Medicine, Research Institute for Convergence of Biomediocal Science and Technology, Pusan National University Yangsan Hospital, 20-Geumo-ro, Mulgeum-eup, Yangsan, Gyeongsangnam-do 50612 South Korea; 3 Department of Neurology, Pusan National University Yangsan Hospital, Pusan National University School of Medicine, Research Institute for Convergence of Biomediocal Science and Technology, Pusan National University Yangsan Hospital, Yangsan, South Korea

**Keywords:** Ocular motility disorders, Peripheral neuropathies

## Abstract

This retrospective study aimed to compare objective/subjective torsion and other clinical characteristics of patients with acquired trochlear nerve palsy. This study included 82 consecutive patients who were diagnosed with acquired fourth cranial nerve palsy between 2014 and 2021 and who were followed up for ≥ 6 months. The etiologies, ocular deviation, objective and subjective torsions were reviewed. The etiologies were classified as ischemic, traumatic, brain lesion, idiopathic, or other. The patients were classified into two groups according to the recovery state: full recovery and partial/no-recovery. We compared the torsion and clinical features based on the etiology and recovery state. The average age was 59.1 ± 11.1 years, and 58 (71.0%) of the patients were male. The most common cause was ischemic (n = 49, 59.7%) and other common causes included traumatic (n = 16, 19.5%), brain lesion (n = 8, 9.8%), idiopathic (n = 5, 6.1%) and others (n = 4, 4.9%). Of the 82 patients, 56 (68.3%) were assigned to the full recovery group, and 26 (31.7%) were assigned to the partial/no-recovery group. The average age and number of patients with ischemic causes of palsy were greater in the full recovery group (*p* = 0.026 and *p* < 0.000, respectively). The vertical deviation angle, tilted angle on the Lancaster red-green test (LRGT), proportion of patients who experienced subjective torsion on the LRGT, and head tilt were smaller in the full recovery group (*p* = 0.037, 0.042, 0.045, and 0.006, respectively). Ischemic trochlear nerve palsy, advanced age, a small deviation angle at the primary position, and few cases of excyclotorsion on LRGT were characteristic of the full recovery group of acquired unilateral trochlear nerve palsy patients.

## Introduction

Trochlear nerve (fourth cranial nerve, CN4) palsy is the most common cause of acquired vertical diplopia^1^, which causes cyclodeviation or ocular torsion, leading to the perception of image tilting^2^. Several studies have reported that decompensation of congenital CN4 palsy is the most common cause of acute vertical diplopia^3,4^, while a vascular etiology is the main cause of acquired cranial nerve palsy^5–9^.

The recovery of diplopia caused by cranial nerve palsy is related to the etiology of the palsy. Ischemic palsy is reported to have a better outcome^5^. Recent studies reported that patients who had larger vertical angles of deviation, severe ocular limitations, intracranial masses, large fundus excyclotorsions, and more frequent head tilting did not fully recover^5,6^. However, the former study^5^ included the decompensation of congenital palsy and the latter^6^ did not include subjective torsion with the Maddox double rod test (MDRT).

We aimed to compare the objective torsion on fundus photography, the subjective torsion on MDRT, the Lancaster red-green test (LRGT), and other clinical characteristics according to the etiology and recovery state.

## Subjects and methods

### Subjects

This study was approved by the Institutional Review Board of Pusan National University Yangsan Hospital (IRB no.: 05-2022-279) and was conducted according to the tenets of the Declaration of Helsinki. The medical records of patients who were diagnosed with acquired unilateral CN4 palsy between 2014 and 2021 and who were followed up for more than 6 months, were retrospectively reviewed. Patients who were followed up for less than 6 months with complete recovery were also included.

CN4 palsy was diagnosed based on the clinical presentation, which included hypertropia at the primary position. This hypertropia increased during opposite side lateral gaze and a same side head tilt, accompanied by over-elevation in adduction in patients presenting with acute vertical diplopia. Patients who were previously diagnosed with paralytic or restrictive strabismus or who had undergone orbital or extraocular muscle surgery were excluded. Patients with congenital or decompensated longstanding CN4 palsy were also excluded. Patients with bilateral CN palsies were excluded; The exclusion criteria were cyclotorsion > 10 degrees on the MDRT and/or bilateral over-elevation in adduction.

The patients’ age of onset, sex, presence of head tilt, findings from brain magnetic resonance imaging (MRI), and previous medical history, especially the presence of vascular risk factors (hypertension, diabetes mellitus [DM], dyslipidemia, and coronary vascular disease), were reviewed together with other neurological symptoms.

The patients were classified into five categories according to etiology: ischemic, traumatic, brain lesion, idiopathic, and others. The ischemic group had at least one vascular risk factor, including hypertension, DM, dyslipidemia, and coronary vascular disease, without a history of trauma or evidence of brain lesions on imaging studies. If microangiopathy or other vascular abnormalities were observed on brain imaging, the patient also assigned to the ischemic group. Those who had an apparent history of head trauma before symptom onset were classified into the traumatic group. If an aneurysm, cerebral hemorrhage, or brain tumor was observed on MRI, the patient was assigned to the brain lesion group. Other causes included complications of other anatomical lesions and inflammation or a systemic disease that affected cranial nerve function. If any of the etiologic criteria listed above were not met, the condition was defined as idiopathic CN4 palsy.

All the patients underwent a complete ophthalmic examination. The angle of ocular deviation was measured using a prism and alternative cover test at the primary, secondary, and tilting positions while fixing at both 6 m and 1/3 m. Ductions were evaluated simultaneously. Superior and inferior oblique muscle dysfunctions were graded on a scale of − 4 to + 4^10^. Objective cyclotorsion was assessed using fundus photography, and subjective torsion was assessed with the LRGT and MDRT.

Full recovery was defined as the absence of vertical deviation, an ocular motor limitation, and vertical diplopia. Partial recovery was defined as a decrease by more than 50% of the initial deviation angle. No-recovery was defined as the absence of full or partial recovery, or cases that underwent extraocular muscle surgery for recovery.

### Statistical analysis

Pearson’s chi-square test was used to evaluate categorical variables. Fisher’s exact test was performed to evaluate categorical variables for which more than 20% of the cells had fewer than 5. Independent Student’s t-tests were used to compare continuous numerical variables. Before performing the t-tests, normality checks were conducted. All *p* values provided were obtained using a two-tailed test. SPSS version 20.0 (SPSS Inc., Chicago, IL, USA) was used for the analysis. *P* values < 0.05 were considered statistically significant.

### Ethical approval and informed consent

Informed consent was obtained from all individual patients included in the study.

## Results

A total of 82 patients were included in this study. The mean follow up period was 7.7 ± 3.9 months. The average age at the first visit was 59.1 ± 11.1 years. There were 58 (71.0%) men and 24 (29.0%) women. None of the patients had accompanying pain. Etiologies were classified into ischemic (n = 49, 59.7%), traumatic (n = 16, 19.5%), brain lesion (n = 8, 9.8%), idiopathic (n = 5, 6.1%), and other (n = 4, 4.9%) categories. Objective excyclotorsion on fundus photography was observed in 65 (79.3%) patients, while subjective excyclotorsion on MDRT and LRGT were found in 58 (70.7%) and 51 (62.2%) patients, respectively. Among the 82 patients, 23 (28.0%) exhibited hypertension, 15 (18.3%) had DM, and 29 (35.4%) had dyslipidemia (Table 1).

**Table 1:** Demographics of patients with fourth cranial nerve palsy.

Parameters	
Age (years)	59.1 ± 11.1
Sex	
Male : Female (n, %)	58 (70.7):24 (29.3)
Follow up period (months)	7.7 ± 3.9
Full recovery (n, %)	56 (68.3)
Duration from onset to recovery (months)	3.6 ± 2.4
Partial recovery (n, %)	7 (8.5)
No recovery	19 (23.2)
Etiology	
Ischemic	49 (59.7)
Traumatic	16 (19.5)
Brain lesion	8 (9.8)
Others (MG, THS, Viral)	4 (4.9)
Idiopathic	5 (6.1)
Deviation angle at primary position (PD)	5.05 ± 3.48
Presence of excyclotorsion (n, %)	
Excyclotorsion on fundus photo (n, %)	65 (79.3)
Maddox double rod test (n, %)	58 (70.7)
Lancaster red-green test (n, %)	51 (62.2)
Degree of excyclotorsion	
Tilted angle on fundus photo (degree)	9.68 ± 4.07
Tilted angle on Maddox double rod test (degree)	6.44 ± 5.51
Tilted angle on Lancaster test (degree)	9.8 ± 9.2
Presence of a contralateral head tilt (n, %)	22 (26.8)
Presence of other neurological symptoms (n, %)	10 (12.2)
Medical history	
Hypertension (n, %)	23 (28.0)
Diabetic mellitus (n, %)	15 (18.3)
Dyslipidemia (n, %)	29 (35.4)

The age at diagnosis was lower in the traumatic CN4 group than in the other etiologic groups. The traumatic group had the longest total follow-up period. Furthermore, in the traumatic group, the number of patients with excyclotorsion on fundus photography, MDRT, and LRGT was greater than that in the other etiologies group. The number of patients who had medical histories, including DM, hypertension or dyslipidemia was greater in the ischemic group. Other neurologic symptoms, such as visual field defects or other cranial nerve palsy, were frequently associated with the brain lesion group (Table 2).

**Table 2:** Comparison of clinical characteristics and manifestations according to recovery state.

Parameters	Ischemic	Traumatic	Brain lesions	*Others*	*Idiopathic*
Numbers of patients (n,%)	49 (59.7)	16 (19.5)	8 (9.8)	4 (4.9)	5 (6.1)
Age (years)	63.4 ± 9.8	46 ± 7.92	60.3 ± 12.4	58 ± 10.8	59 ± 9.8
Sex (Male : Female)	35 : 14	13 : 3	5:3	2:2	3:2
Total follow up period (months)	3.4 ± 2.3	24.1 ± 19.8	15.3 ± 6.7	4.2 ± 2.8	3.7 ± 2.6
Complete recovery (n, %)	43 (87.8)	5 (31.3)	1 (12.5)	3 (75.0)	4 (80.0)
Duration from onset to recovery (months)	2.9 ± 1.7	7.9 ± 5.5	2.0	3.8 ± 2.7	3.2 ± 2.3
Deviation angle at primary position (PD)	4.6 ± 3.61	7.1 ± 3.8	5.0 ± 3.4	4.2 ± 2.3	3.8 ± 1.1
Presence of excyclotorsion					
Excyclotorsion on fundus photo (n, %)	39 (79.6)	16 (100)	6 (75.0)	2 (50.0)	2 (40.0)
Maddox double rod test (n, %)	33 (67.3)	16 (100)	5 (62.5)	2 (50.0)	2 (40.0)
Lancaster red-green test (n, %)	29 (59.2)	16 (100)	4 (50.0)	1 (25.0)	1 (20.0)
Degree of excyclotorsion					
Tilted angle on fundus photo (degree)	9.2 ± 3.81	12.17 ± 2.79	9.5 ± 4.12	8.8 ± 4.89	7.9 ± 3.87
Tilted angle on Maddox double rod test (degree)	5.44 ± 5.51	9.8 ± 6.7	5.6 ± 4.3	7.2 ± 5.2	6.5 ± 4.8
Tilted angle on Lancaster test (degree)	8.1 ± 8.95	17 ± 14.51	10.5 ± 6.6	4.9 ± 3.5	4.5 ± 3.6
Presence of a contralateral head tilt (n, %)	10 (20.4)	9 (56.3)	1 (12.5)	1 (25.0)	1 (20.0)
Presence of other neurological symptoms (n, %)	0 (0)	2 (12.5)	8 (100)	0 (0)	0 (0)
Medical history					
Hypertension (n, %)	20 (40.8)	2		1	0
Diabetic mellitus (n, %)	15 (30.6)	0	0	0	0
Dyslipidemia (n, %)	27 (55.1)	1	1	0	0

Full recovery was observed in 56 patients (68.3%), with a mean duration of recovery of 2.6 ± 2.4 months. Partial recovery was observed in 7 patients (8.5%), and 19 patients (23.2%) did not recover. The clinical characteristics of patients in the full recovery and partial/no recovery groups were compared. Patients in the full recovery group were older than those in the partial/no recovery group (61.53 ± 9.6 vs. 53.96 ± 13.51, *p* = 0.026). The etiologies were significantly different between the two groups (*p* < 0.001). The full recovery group tended to have ischemic CN4 palsy, whereas the partial/no recovery group tended to have traumatic and brain lesion CN4 palsy. The angles of vertical deviation were significantly greater in the partial/no recovery group than in the full recovery group (*p* = 0.037). The number of patients who had excyclotorsion on fundus photography and MDRT did not differ between the two groups. The number of patients with excyclotorsion on the LRGT was greater in the partial/no recovery group than in the full recovery group (*p* = 0.045). The degree of tilted angles on the fundus photography and the MDRT did not differ between the two groups (*p* = 0.172 and 0.193). A greater angle of excyclotorsion on the LRGT was observed in the partial/no recovery group (*p* = 0.042). The number of patients who had a head tilt to the contralateral side was greater in the partial/no recovery group (*p* = 0.006) (Table 3).

**Table 3:** Comparison of clinical characteristics and manifestations according to etiology.

Parameters	Full recovery	Incomplete recovery	*p*
Numbers of patients	56 (68.3)	26 (31.7)	
Age (years)	61.53 ± 9.6	53.96 ± 13.51	0.026*
Sex (Male : Female)	38:18	20:6	0.705^†^
Etiology			
Ischemic	43 (76.8)	6 (23.1)	< 0.0001^††^
Traumatic	5 (8.9)	11 (42.3)	
Brain lesions	1 (17.8)	7 (26.9)	
Others	3 (5.4)	1 (3.8)	
Idiopathic	4 (7.1)	1 (3.8)	
Deviation angle at primary position (PD)	4.75 ± 3.74	5.91 ± 2.79	0.037*
Presence of excyclotorsion			
Torsion on fundus photo (n, %)	40 (71.4)	25 (96.1)	0.391^†^
Maddox double rod test (n, %)	35 (62.5)	23 (88.5)	0.334^†^
Lancaster red-green test (n, %)	26 (46.4)	25 (96.2)	0.045^†^
Degree of excyclotorsion			
Tilted angle on fundus photo (degree)	9.45 ± 3.8	10.8 ± 4.6	0.172*
Tilted angle on Maddox double rod test (degree)	6.0 ± 5.12	7.43 ± 4.85	0.193*
Tilted angle on Lancaster test (degree)	8.51 ± 8.59	12.25 ± 10.93	0.042*
Presence of a contralateral head tilt (n, %)	8 (14.3)	14 (53.8)	0.006^†^
Presence of other neurological symptoms (n, %)	4 (7.1)	6 (23.1)	0.076^†^

*Comparison between the groups by Student’s *t*-test.

^†^Comparison between the groups by Pearson’s chi-square test.

^††^Comparison between the groups by Fisher’s exact test.

## Discussion

In this study, patients who had acquired CN4 palsy due to an ischemic cause recovered within 2–4 months, and more older patients were assigned to the full recovery group. The degree of vertical misalignment and excyclotorsion of CN4 palsy was significantly greater in the partial/no recovery group than in the full recovery group.

Acquired third, fourth, and sixth cranial nerve palsies are likely to be early indications of brain lesions, and thus require an immediate diagnosis^11^. Pupils involved in oculomotor nerve palsy must be urgently evaluated, as these symptoms may indicate an emergency condition such as an aneurysm. Pupil-sparing oculomotor nerve palsy is often caused by microvascular ischemia^12^. The most common etiology of sixth cranial nerve palsy is vascular disease, but other causes include idiopathic, intracranial neoplasm, trauma, cerebral aneurysm, and intracranial inflammation or infection^13^.

Several studies have reported different results concerning the frequency of the different causes of trochlear nerve palsy. The most commonly reported type of trochlear nerve palsy is congenital palsy and traumatic causes are the second most common type^9,14^. Richards et al.^3^ reported that out of 657 patients with acquired CN4 palsy, 28% had an undetermined etiology, 25% had head trauma, and 15% had vascular disease. This is because CN4 is more vulnerable to a traumatic injury than the third and sixth cranial nerve due to its long course, posterior decussation, and slender anatomy^7^. On the other hand, Park et al.^4^ reviewed 46 cases of CN4 palsy, and 37% of patients had a vascular cause and 30% of patients had traumatic causes. Oh et al. reported that 60% of CN4 palsy patients had a vascular etiology, and 21.3% of patients had a history of trauma^5^. In this study, ischemic CN4 palsy was the most common, with 59.7% of cases being due to ischemic causes and 19.5% due to traumatic causes. These results can be explained by the different referral patterns based on the population distribution.

There is a longstanding debate about the clinical definition of microvascular ocular motor nerve infarction. Complete resolution within 6 months seems to be a widely accepted criterion along with the presence of vascular risk factors as reported by many authors^15^. This study included microvascular cases that had not been completely resolved within 6 months, and there is a potential for overdiagnosis. In this study, the proportion of idiopathic cases was notably low, at 6%, compared to that in other reports^3,16^. However, in recent studies, the proportion of idiopathic cases has been reported to be 2.9–3.75%, similar to the results of our study^5,6^. This could be due to the inclusion of patients in the ischemic etiology group who had microangiopathy on MRI, and the inclusion of these patients increased the proportion of ischemic cases.

Recovery rates ranging from 40 to 85% have been reported in previous studies on trochlear nerve palsy^4,5,14^. The full recovery rate in this study was 68.3%. Among other etiologies, vascular causes of trochlear nerve palsy were associated with a significantly greater recovery rate. The recovery rate of ischemic trochlear nerve palsy has been reported to be 75–93.5%^8,17^. In our study, the full recovery rate of patients with ischemic CN4 palsy was 87.8%, and the duration of recovery was 2.9 months, suggesting that most ischemic CN palsy patients achieved complete recovery within 3 months. These results are similar, considering that, in another study, 93.5% of presumed microvascular CN4 palsy cases were resolved within 2–3 months^8^. The recovery rate of traumatic trochlear nerve palsy patients has been reported to be lower, ranging from 50 to − 64.7%, than that of ischemic trochlear nerve palsy patients^5,6^. In this study, the full recovery rate of patients with traumatic CN4 palsy was 31.3%.

Previous studies reported that 43.8–50% of patients with a vascular etiology had hypertension^5,8^. In this study, 40.8%, 30.6%, and 55.1% of patients had hypertension, DM, and dyslipidemia, respectively.

Ischemic CN4 palsy (76.8%) was the most common cause in patients who achieved full recovery, whereas traumatic (42.3%) and brain lesion (26.9%) CN4 palsy accounted for 69.2% of all patients in the partial/no-recovery group. The full recovery rate of patients with traumatic CN4 palsy was 31.3%, and the duration of recovery was 7.9 months. Only one of the eight patients with CN4 palsy caused by a brain lesion fully recovered after vertical diplopia. The patient had CN palsy due to pituitary apoplexy and recovered within 2 months after tumor removal. Among seven patients, six had cavernous invasion of a tumor, and one had a clinoid aneurysm. They did not recover after 1 year. The recovery rate varies depending on the etiology, the cause of the palsy can be used as a factor to predict the prognosis.

A larger vertical deviation has been reported to be a risk factor for incomplete recovery^4,5^. Park et al.^4^ reported that a larger angle of deviation in ocular motor palsy patients is related to a worse prognosis. Oh et al.^5^ reported that the initial vertical deviation angle at the primary or ipsilateral tilt position was smaller in patients who achieved complete recovery from trochlear nerve palsy. These findings are explained by the fact that the angle of deviation in patients with CN4 palsy with a vascular cause is smaller than that of patients with CN palsy due to other causes of trauma or a brain lesion and that the most common etiology in the complete recovery group was a vascular cause. In this study, the initial vertical deviation was smaller in the full recovery group than in the partial/no-recovery group, similar to the findings of other studies^4,5^. Furthermore, a head tilt was more common in the partial/no-recovery group, similar to the findings of other studies^5^, and most of the patients with a distinctive head tilt experienced sustained trauma and brain lesions.

The detection of extorsion and the amount of extorsion measured by the LRGT, fundus photography, and the MDRT were highly diverse. Roh and Hwang^18^ reported that the most sensitive method was fundus photography (100%), followed by MDRT (91%), and LRGT (46%). In this study, 65% of the extorsions were detected via fundus photography, 58% via the MDRT, and 51% via the LRGT. The fundus extorsion was reported to be more frequent and larger in patients with poorer outcomes^5,6^. Further subjective torsion by LRGT was reported as a possible prognostic factor for poor prognosis^6^. In this study, the frequency and amount of objective torsion on fundus photography did not differ between the two groups. The frequency and the amount of excyclotorsion based on the MDRT did not differ between the two groups. The number of patients with excyclotorsion based on the LRGT was greater in the partial/no-recovery group than in the full recovery group. This is because the partial/no-recovery group included a greater number of patients with traumatic causes of trochlear nerve palsy, and the amount of torsion was large in patients with traumatic cause.

Our study has several limitations. First, this was a retrospective single hospital-based study, resulting in selection bias, and the outcomes may differ according to the population or geographic location. Second, the differential diagnosis of vascular and idiopathic etiologies may be inaccurate because they are based on the presence of vascular risk factors and MRI findings. Third, an upright-supine test was not performed to confirm the preservation of the otolith-ocular pathways. Differentiating a case of skew deviation with incomitant torsion from a case of isolated CN4 palsy may be inaccurate. Fourth, LRGT was performed using a traditional grid with a foster torch. Therefore, it was difficult to accurately and quantitatively record the mangnitude of torsion. Nonetheless, one advantage of this study is that only evaluated objective/subjective torsion in acquired unilateral CN4 palsy patients with acute vertical diplopia.

In conclusion, our investigation revealed that the most common cause of acquired CN4 palsy was an ischemic etiology, and traumatic causes were the second most common cause. The overall full recovery rate from acquired CN4 palsy was 68.3%. Ischemic causes of CN4 palsy and old age were characteristics of the full recovery group. The vertical misalignment and excyclotorsion on the LRGT were significantly more prominent in the partial/no recovery group.

## Data Availability

The datasets used and/or analyzed during the current study available from the corresponding author on reasonable request.
